# Ascorbate inhibition of angiogenesis in aortic rings ex vivo and subcutaneous Matrigel plugs in vivo

**DOI:** 10.1186/2040-2384-2-2

**Published:** 2010-01-18

**Authors:** Nina A Mikirova, Joseph J Casciari, Neil H Riordan

**Affiliations:** 1 Bio-Communications Research Institute, Wichita, Kansas, USA

## Abstract

**Background:**

Angiogenesis is critical to tumor growth and is therefore a potential target for cancer therapy. As many current inhibitors of angiogenesis exhibit host toxicity, natural alternatives are needed. At millimolar concentrations, ascorbate (vitamin C) inhibits migration and tubule formation by mature endothelial cells and endothelial progenitors. In the present study, we examined the effects of ascorbate, at levels relevant during intravenous infusion therapy, on angiogenesis using an ex vivo an in vivo assay.

**Methods:**

Two assays were used to evaluate effect of high-doses ascorbic acid on angiogenesis: ex vivo rat aortic ring explant assay in Matrigel matrices and in vivo Matrigel plug assay. In aortic rings, we quantified microvessel growth, branching and vessel regression under different treatment conditions. In murine angiogenesis assay, male C57 mice 6-8 weeks old were treated by high-dose ascorbic acid and the number of microvessels was analyzed by histological method. To characterize the population of cells that formed capillary network and microvessels, the sections were stained by CD34 and CD31 antibodies.

**Results:**

Results show that sprouting of endothelial tubules from aortic rings was reduced in a concentration-dependent fashion by ascorbate: while controls roughly tripled sprout densities during the study, ascorbate (1 mg/mL, 5.5 mM) actually reduced sprout density. In vivo, the ability of mice to vascularize subcutaneously implanted Matrigel plug was diminished if the mice were treated with 430 mg/kg vitamin C: numbers of vessels, and vessel densities, in plugs from treated mice were roughly 30% less than those in plugs from untreated mice.

**Conclusions:**

We conclude that the inhibition of angiogenesis by ascorbate suggested in vitro is confirmed in vivo, and that angiogenesis inhibition may be one mechanism by which intravenous ascorbate therapy shows efficacy in animal experiments and clinical case studies.

## Background

Angiogenesis, the generation of new blood vessels, is thought to be necessary for tumor growth and metastasis [1-5]. Thus, there is considerable interest in developing anti-cancer therapies based on the inhibition of tumor-induced angiogenesis [6,7]. Most angiogenesis inhibitors currently being tested work either by neutralizing endothelial cell growth factors, inhibition endothelial cell proliferation, preventing turnover of basement membrane, or blocking capillary formation. Unfortunately, most of these agents are toxic and have high risk of adverse effects. In hopes of finding less toxic angiogenesis inhibitors, substances derived from natural sources, such as flavonoids, sulphated carbohydrates, or triterpenoids are being examined, as are natural health products such as herbs, phytochemicals, and antioxidants [8,9].

In regard to antioxidant angiogenesis inhibitors, vitamin C is of particular interest for a variety of reasons. First, it is has already been shown to have anti-tumor effects in certain experimental and clinical settings, provided that ascorbate concentrations reach the millimolar range [10-15]. Secondly, its role in supporting the later stages of wound healing, particularly collagen formation and strengthening of extracellular matrix, may counter tumor-induced neovascularization [10]. Moreover, its antioxidant activity may regulate some aspects of endothelial cell migration, since some aspects of this are promoted by nitric oxide and other oxidants [16].

In a recent study, sodium ascorbate at millimolar concentrations was shown to inhibit tubule formation by endothelial cells on Matrigel, endothelial cell proliferation (but not viability), endothelial cell migration, and reduce nitric oxide concentrations (NO is a major stimulator of angiogenesis). These observations held for both mature endothelial cells and endothelial progenitor cells [16].

The previous work is limited to study of in vitro models. This typically raises two questions: do the observations extend to a more physiological model system, and are the pharmacokinetics of vitamin C such that sufficient levels can be attained in situ to reproduce in vitro effects. The goal of the present manuscript is to test the hypothesis that ascorbate, delivered by injection at doses designed to attain millimolar plasma concentrations, will inhibit neovascularization in vivo. To study a more physiological model (using tissue sections rather than cell lines), we examined the effect of ascorbate on outgrowth of microvessels from rat aorta rings. To address the various issues associated with in vivo application, we examined the effect of ascorbate injections on the growth of new blood vessels into Matrigel plugs implanted into mice. This latter model provides an excellent test of in vivo angiogenesis when agent biodistribution is taken into account.

## Methods

### Aortic ring assay

Rat aortic ring explant cultures were prepared by modification of protocols previously described [17-21]. 24 well plates were covered by 150 ul of Matrigel (Becton Dickenson, Bedford, MA). Aortic rings were prepared from male Sprague Dawley rats. Aortas were sectioned into 1 mm-long cross sections, rinsed several times with endothelial growth medium M-200 (Cascade Biologics), placed on Matrigel in wells and covered with an additional 50 ul of Matrigel. The rings were cultured in 1 ml of endothelial medium for several days. Cultures were incubated at 37C, in humidified CO_2 _and the medium was replaced daily. As microvessels began to branch and develop, some wells with aortic rings were kept as control and other wells were treated with sodium ascorbic acid. Analysis of the macrovessel growth was performed by counting number of pixels by NIH Image software program (NIH, Bethesda, MD) [22].

### Matrigel plug assay

In vivo angiogenesis measurements using Matrigel plugs were performed as previously described [23,24]. Normal male mice (C57BL/6) 6-8 weeks of age and weighing ~20 g were obtained from Charles River Laboratory and allowed to acclimate for 3-4 days prior to the start of treatments. C57Bl/6 mice were used as a host, since the EHB tumor (i.e. Endelbreth-Holm-Swarm sarcoma) from which Matrigel is derived, is propagated in these mice and therefore does not elicit an immune response and does not require the use of athymic mice. The study was approved by the Institutional Review Board and was conducted in accordance with the Declaration of Helsinki and the Office of Laboratory Animal Welfare regulation. Aliquots of Matrigel with growth factors (FGF and VEGF) were prepared on ice. Final concentrations of growth factors used in all experiments were 50 ng/ml FGF and 100 ng/ml VEGF. Matrigel (0.8 mL) was injected into the ventral side of mouse, as it is the best area in terms of angiogenic response. Matrigel was allowed to gel for about 10 sec before removing needle at the end of injection.

Sodium ascorbate was administered every other day for a period of two weeks, the alternate day cycle being consistent with how intravenous vitamin C is used clinically at our facility [14,15]. Fresh sodium ascorbic acid solution was prepared every other day, and a dosage of 8.6 mg per mouse (430 mg/kg for a 20 gram mouse) was used (in clinical studies with humans, this dosage was sufficient to give plasma ascorbate concentrations in the millimolar range). We found in preliminary studies that two weeks was a sufficient time period to allow measurable neovascularization of the implanted Matrigel plug. After two weeks of treatment, mice were euthanized by CO_2 _asphyxiation for plug exclusion. Matrigel plugs were removed two weeks after implantation and were fixed in 10% neutral buffered formalin prior to paraffin processing. These experiments have been reproduced three times.

The angiogenic activity was analyzed by immunohistochemical staining and staining by hematoxylin and eosin. Total number of microvessels containing red blood cells and number of capillaries in a high power field (400× magnifications) were counted. Vessel count and size was determined using ProgRes Capture Pro software.

To further analyze these microvessels, some sections were stained with antibodies to CD34 (rabbit polyclonal, Abcam) and CD31 (1:200, goat poly-IgG, Santa Cruz). Antibodies were detected using 1% hydrogen peroxide followed by avidin-biotin complex (ABC kit, Vector Laboratories) and visualized with diaminobenzidine (DAB).

### Statistical analysis

For comparison of microvessel densities and capillary densities for treated and non-treated groups, statistical analysis was carried out by Mann-Whitney U test. P values less than 0.05 were considered significant.

## Results

**Figure 1:**
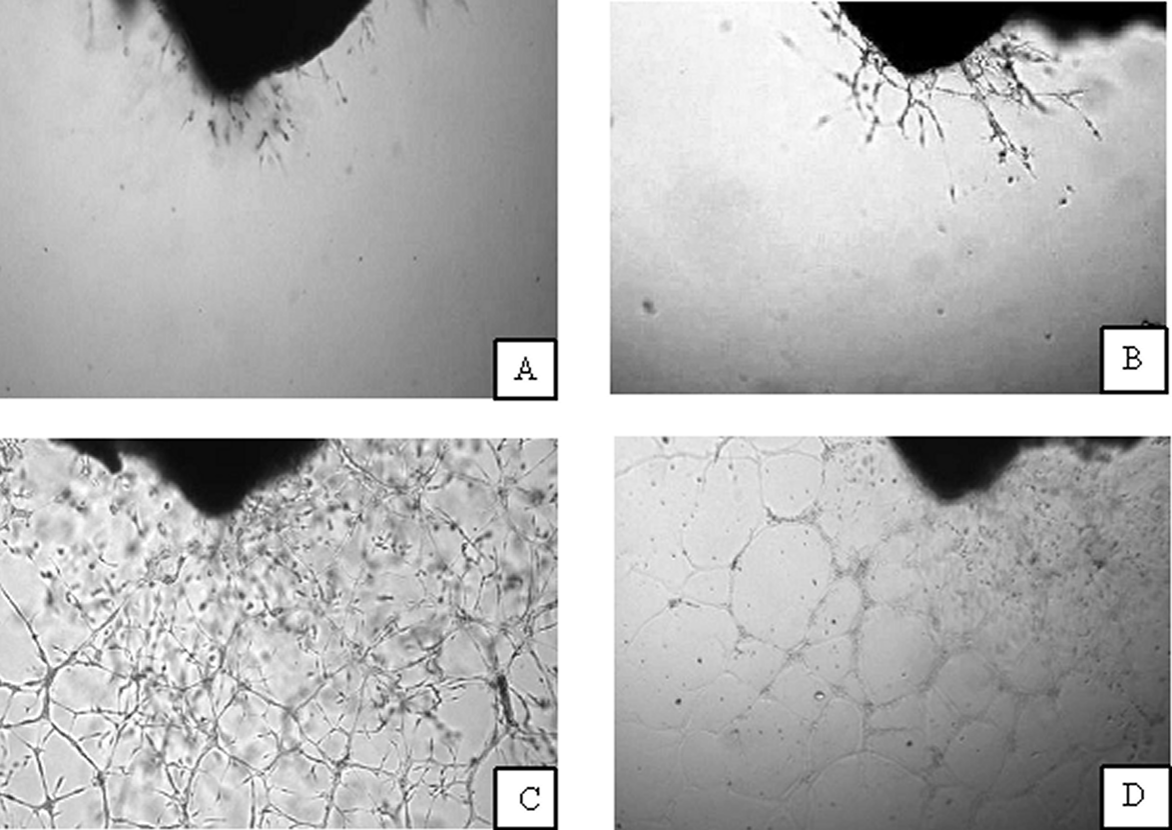
**Developing microvessels from the intimal/subintimal layers of the aortic wall**. A, B-microvessel outgrowth at the beginning of experiments, C-non-treated well, D-well treated by 1 mg/mL ascorbic acid during 4 days.

The outgrowths of endothelial tubules from aorta rings are shown in Figure 1. Panels A and C show untreated controls at days 0 and 4, respectively. There is an obvious expansion of tubules, presumably a combination of endothelial cells, mesenchymal cells, and perivascular cells [25-27]. In contrast, this expansion was suppressed by incubation in 1 mg/mL (5 mM) ascorbic acid: Panels B and D show ascorbate treated rings at days 0 and 4, respectively. As a further check, we analyzed the sprouting using the higher magnification and found that there was additional vessel development from aortic intimal/subintimal layers. Thus, angiogenesis as observed in our analysis was due to new vessel formation and not just sprouting from the initial vessels. By starting treatment when we saw initial vessel development, we excluded samples, in which vessel development was inhibited for other reasons.

**Figure 2:**
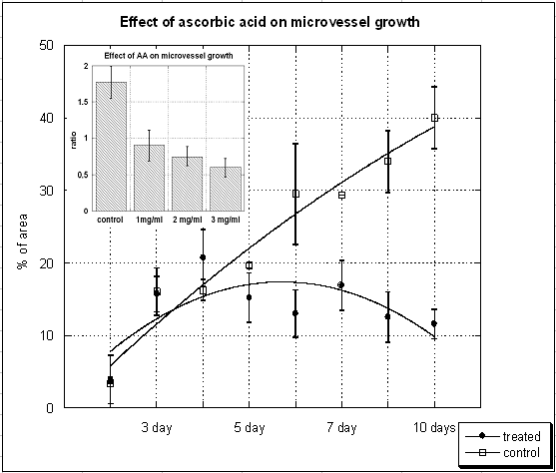
**The effect of ascorbic acid on microvessel growth in aortic ring assay**. Microvessel outgrowths (percentage of area occupied by vessels) for controls and treated aorta rings as a function of time. Ascorbate was administered starting on day 3 at a concentration of 1 mg/dL (5 mM) (lines represent second order polynomial fits via Kaleidograph). The inset shows ratios of microvessel areas before and after treatment for five days rings exposed with various levels of ascorbate.

Figure 2 shows the effect of ascorbate on the percentage of total area in the field of view occupied by tubules. Outgrowth area (percent total) as a function of time and of dosage (inset, after 5 days of treatment) is shown. Ascorbate was administered starting on day three at a concentration of 1 mg/dL (5 mM). The inset shows ratios of microvessel areas before and after treatment for five days rings exposed with various levels of ascorbate. While the growth of endothelial microvessels from the aorta continues steadily in controls, new formations stop forming by day two in ascorbate treated samples. As shown in the bar graph inset, there is a dose-response effect, with higher levels of ascorbate leading to reduced outgrowth of vascular shoots. The levels of vitamin C used here are consistent with those used in plasma during ascorbate injection therapy [28,29]. Hence, the inhibition of angiogenesis seen here is occurring under conditions that may be relevant during treatment.

Histological sections of Matrigel plugs were examined. The hematoxylin/eosin stains of cross sections of the plugs demonstrated that injection of Matrigel with VEGF and bFGF to the mice induced cellularity, the formation of tubules and many blood-filled channels containing red blood cells. We saw both large vessels, presumably the result of preexisting vessels extending into the Matrigel, and microvessels and capillaries that may be examples of angiogenesis and neovascularization. To characterize the population of cells that formed capillary network and microvessels, the sections were stained by markers that demonstrated the endothelial type of cells. Matrigel plugs were stained antibodies to CD34 and CD31; positive cells are seen in both cases, demonstrating that the vessels formed within these plugs are endothelial cell origin.

**Figure 3:**
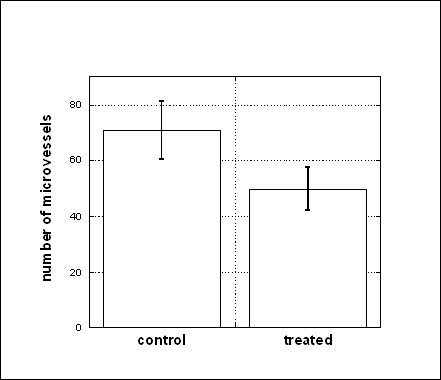
**The effect of ascorbic acid treatment of the number of microvessels in Matrigel plugs**. Number of microvessels (average ± SE) per Matrigel plug section for controls and for mice treated. Statistical analysis of the data were done using the Mann-Whitney U test and demonstrated the difference in microvessel formation in control and treated mice (p < 0.05).

The effect of vitamin C on vascularization in Matrigel plugs in vivo can be quantified by counting total numbers of microvessels using 200× magnification. Results are shown in Figure 3. The decrease in microvessel formation concurrent with ascorbate therapy (70 ± 25 for control and 49 ± 20 for treated mice) and is statistically significant (one-sided p-value < 0.05). This was verified by capillary density measurements using image software: Matrigel plugs from control mice showed capillary densities of 30% ± 6%, while those from ascorbate treated mice showed capillary densities of 22% ± 5%. In short, subcutaneous injections of Matrigel in mice induce plugs containing new vessels, comprised in part of endothelial and progenitor cells. This neovascularization is diminished in mice treated with ascorbate at doses used in clinical studies with intravenous infusions.

## Discussion

The results detailed above, along with those in our previous publication, suggest that ascorbate has an inhibitory effect on angiogenesis and neovascularization at concentrations relevant to those attained in intravenous ascorbate therapy. Our previous in vitro studies [16] showed that ascorbate inhibited several aspects of angiogenesis that are observable in vitro (tubule formation on Matrigel, migration to fill gaps on monolayer, and endothelial cell proliferation); moreover, these effects were more pronounced on new vessel formation than on existing vessels.

Experiments in aortic rings allowed examination of ascorbate effects under conditions where endothelial cells, progenitor cells, and other micro environmental factors are interacting. Vitamin C is likely to regulate, or impact, several processes of various cell types present. The overall effect, however, is inhibitory. The Matrigel plug assay allowed us to examine ascorbate in an in vivo environment. This is important not only because of the complexities involved in neovascularization in situ, but also because of concerns about ascorbate pharmacokinetics. In these studies, ascorbate clearly had an inhibitory effect, suggesting that it was distributed to these tissues in sufficient quantities to act. One complication to interpretation of these results is that mice are able to produce their own vitamin C, an ability not shared by humans. Ascorbate levels were not measured in our Matrigel plugs, so we do not know what the actual concentrations were at the angiogenesis site. It is possible that neovascularization in control mice was less than it could have been because the mice produced extra ascorbate in response to the stress of participating in the study.

The mechanism of action by which vitamin C may inhibit neovascularization is still subject to speculation. Earlier work suggests that ascorbate may act by reducing nitric oxide levels; as NO is an important regulator of angiogenesis [16]. Ascorbate may inhibit angiogenesis as an antioxidant by suppressing angiogenesis stimulated by reactive oxygen species [30]. For example, the scavenging of peroxynitrite reduces vascular permeability in tumor [31].

In the situation of tumor angiogenesis, vitamin C may possibly have other beneficial effects. The inhibitory effect of ascorbate in tube formation is involved in the preferentially enhanced type I and III collagen synthesis [32]. Ascorbate can have direct cytotoxicity against tumor cells (which in turn would reduce the production of pro-angiogenesis cytokines) in some instances [13,14], and/or it can inhibit the expression of angiogenesis-related genes [33]. This assertion is supported by recent research indicating that tumor-bearing animals treated with ascorbate survive longer than untreated controls, due to the later forming highly invasive tumors that are not observed in treated animals [34].

Ascorbate may also influence angiogenesis through its effects on hypoxia-inducible factor (HIF). HIF is thought to be important in regulating tumor progression and angiogenesis [35-37]. Ascorbate has been shown to diminish HIF levels [38] and inhibit the enhancement of HIF by glucose metabolites [35]. In fact, Gao et al. have indicated that the ability of ascorbate (and other antioxidants) to inhibit tumor formation relates to its effects on HIF, not to its protection of DNA [38].

As more animal studies and clinical trials are conducted with intravenous ascorbate therapy, the evidence of angiogenesis inhibition should be considered when assessing the results of these studies. Also, this work supports the utility of examining natural products and vitamins for potential angiogenesis regulatory activity.

## Competing interests

The authors declare that they have no competing interests.

## Authors' contributions

NM performed experiments and analyzed the data. NR participated in the design of the study. The manuscript was written by NM and JC. All authors read and approved the final manuscript.
